# Nitroxoline inhibits bladder cancer progression by reversing EMT process and enhancing anti-tumor immunity

**DOI:** 10.7150/jca.47025

**Published:** 2020-09-23

**Authors:** Naijin Xu, Wenfeng Lin, Jingkai Sun, Takuya Sadahira, Abai Xu, Masami Watanabe, Kai Guo, Motoo Araki, Gonghui Li, Chunxiao Liu, Yasutomo Nasu, Peng Huang

**Affiliations:** 1 Department of Urology, Okayama University Graduate School of Medicine, Dentistry and Pharmaceutical Sciences, Okayama, Japan; 2 Department of Urology, Sir Run Run Shaw Hospital, Zhejiang University School of Medicine, Hangzhou, China; 3 Department of Urology, Zhujiang Hospital, Southern Medical University, Guangzhou, China; 4 Center for Innovative Clinical Medicine, Okayama University Hospital; 5 Okayama Medical Innovation Center, Okayama University, Okayama, Japan

**Keywords:** Nitroxoline, Bladder cancer, EMT, immunotherapy preclinical model

## Abstract

Nitroxoline is considered to be an effective treatment for the urinary tract infections. Recently, it has been found to be effective against several cancers. However, few studies have examined the anti-tumor activity of nitroxoline in bladder cancer. The purpose of the study was to reveal the possible mechanisms how nitroxoline inhibited bladder cancer progression. *In vitro* assay, we demonstrated that nitroxoline inhibited bladder cancer cell growth and migration in a concentration-related manner. Western blot analysis demonstrated that nitroxoline downregulated the expressions of epithelial mesenchymal transition (EMT)-related proteins. Furthermore, treatment with nitroxoline in the C3H/He mice bladder cancer subcutaneous model resulted in significant inhibition of tumor growth. Moreover, the percentage of myeloid‐derived suppressor cells (MDSC) in peripheral blood cells significantly decreased after treatment of nitroxoline. Taken together, our results suggested that nitroxoline may be used as a potential drug for bladder cancer.

## Introduction

Bladder cancer is the ninth most common malignancies and the most common urinary tract malignancy in the world 1, 2. Bladder urothelial carcinoma is mainly classified into non-muscular invasive bladder cancer (NMIBC) and muscular invasive bladder cancer (MIBC). Approximately 80% of total bladder cancer cases are NMIBC. Transurethral resection of the tumor (TUR) followed by Bacillus Calmette-Guérin (BCG) instillation is a standard treatment option for NMIBC. However, patients with NMIBC have a high recurrence rate and 20-30% will progress to MIBC. For these reasons, new treatment options are needed to prevent recurrence and progression.

Nitroxoline serves as an antibiotic that has been used clinically in Europe for decades and have showed activity against biofilm infections^3^. The current research shows that nitroxoline has a significant inhibitory effect on many solid tumors, such as myeloma, breast cancer, glioma, pancreatic cancer, and prostate cancer, etc.^4^-^11^, but its effect on bladder cancer is poorly known. Multiple mechanisms have been described as possible modes of the antitumor action of nitroxoline, including the activation of cell apoptosis^6^, downregulation of Na/K-ATPase pump and β-catenin^5^, and inhibition of angiogenesis through regulating the function of MetAP2 12. Notably, several studies have demonstrated that nitroxoline inhibits tumor cell migration and invasion by inhibition of cathepsin B activity 13, 14.

Although no study has shown the effect of nitroxoline on immune response, some indirect evidence supports the possibility of an association. Liang, Y.A etc. suggested that clioquinol is an analog of nitroxoline and engaged in regulating the T cell activation, proliferation, and apoptosis by inhibition of NF-κB function 15. Several studies have demonstrated that nitroxoline can specifically inhibit the activity of cathepsin B 14, 16 that may cause the inhibition of lymphocyte proliferation and regulate immune cell apoptosis 17. Thus, we assumed that an association between nitroxoline and immune responses may exist.

The purpose of this study was to investigate the possible mechanism of nitroxoline on bladder cancer. Our data showed that nitroxoline could reduce the cell viability, induce cell apoptosis and inhibit cell migration in bladder cancer. Furthermore, nitroxoline induced *in vivo* tumor growth inhibition and decreased levels of MDSCs in a murine xenograft model of bladder cancer. Our findings may provide novel strategy for bladder cancer treatment.

## Materials and Methods

### Antibodies and reagents

Nitroxoline was obtained from Jiangsu Asieris Pharmaceuticals, Co., Ltd. (Taizhou, Jiangsu, China) and was dissolved in phosphate-buffered saline (PBS). The following antibodies were used for Western blot analysis: N-cadherin (#13116, CST, Billerica, MA, USA), Slug (#9585, CST), MMP-2 (#87809, CST), MMP-9 (ab38898, Abcam, Cambridge, United Kingdom), β-actin (#4970, CST), Bcl-2 (#3498, CST), and cleaved caspase-3 (#9664, CST). The following antibodies were used for immunohistochemistry and flow cytometry: Ki-67 (#12202, CST), CD31 (ab28364, Abcam, Cambridge, United Kingdom), MMP-9 (ab38898, Abcam, Cambridge, United Kingdom), FITC-labeled anti-CD11b antibody (553310, BD Bioscience, San Jose, CA, USA), PE-labeled anti-Gr-1 (553128, BD Bioscience).

### Cell lines and cell culture conditions

Bladder cancer cell lines MBT-2 and J82 were purchased from ATCC Company of the United States. The cells were maintained in DMEM+10% FBS at 37℃ and 5% CO_2_. The adherent cells were digested with trypsin (including EDTA) to form cell suspension, washed twice with PBS, and resuspended with fresh culture medium.

### XTT viability assay

Cells were plated onto 96-well plates at 2×10^3^ per well. Following cell adherence, cells were treated with nitroxoline at concentrations of 0, 1, 5, 10, 20, and 50 μM, respectively. After nitroxoline treatment for different time points, cells were incubated with XTT compound (Roche Diagnostics, Indianapolis, IN, USA) for 4 hours. The OD value of each well was obtained by a microplate reader (model 680; Bio-Rad Laboratories, Inc., Hercules, CA, USA).

### Western blot analysis

Cells were treated with ice-cold lysis buffer containing a protease and phosphatase inhibitor cocktail (#78410, Thermo Scientific, Waltham, MA, USA). Proteins were transferred to PVDF membrane by SDS polyacrylamide gel (Bio-Rad, Hercules, CA, USA) electrophoresis. The membrane was blocked in 5% non-fat milk for 1 h at room temperature, followed by overnight incubation with the primary antibodies at 4°C. After rinsing with TBST, the membranes were incubated with second antibodies at room temperature for 1 h. Proteins were detected by the enhanced chemiluminescence (ECL) kit (Amersham Pharmacia Biotech, Chandler, AZ, USA).

### Analysis of apoptosis with Hoechst 33324 by fluorescence microscopy

Cells were seeded in 6-well culture plates. After overnight incubation, cells were treated with nitroxoline at concentrations of 0, 10, 20 µM, respectively. After treatment for 48 hours, apoptosis-Hoechst 33324 staining kit (H3570, ThermoFisher Scientific) was used to treat cells for 10 minutes. After that, cells were washed twice with PBS for observation under a fluorescence microscope.

### Wound healing assay

Cells were seeded in 6-well plate up to 90% monolayer confluency. The monolayer of cells was wounded across the bottom of the dish using 100 μL pipette tip, and the floating cells were washed using PBS. After treatment with different concentrations of nitroxoline, wound healing was photographed at 0h, 24h. The degree of wound healing was evaluated as this formula: wound healing (%) = (Original wound area -wound area at 24 hour)/original wound area × 100%.

### Migration assay

After treatment with nitroxoline for 24 hours, cells were digested and re-suspended with serum-free culture medium. Cells (5 × 10^4^) were cultured in the upper chambers of transwell champers (8-μm pore size, #3422, Corning, USA); DMEM containing 20% FBS was added to the lower chamber, and the cells were incubated for 24 hours at 37℃. Cells attached to the upper chamber were wiped with cotton swabs and migrated cells on the lower microporous filter membrane were fixed with 4% paraformaldehyde for 15 min, and stained with 0.1% crystal violet for 30 min.

### Subcutaneous implantation of mouse bladder cancer cells into C3H/He mice

1 x 10^6^ MBT-2 cells suspended in 200µL PBS were subcutaneously implanted into the right flank of C3H/He mice. After tumor formation, nitroxoline were administrated by gavage at 15 mg/kg or 60mg/kg per mouse five times a week on days 1, 2, 4, 5, 7, 8, 10, and 11. The tumor growth was measured with a caliper every 2~3 days. Tumor weights were evaluated on day 12 after the treatments. Animal experimental procedures were approved by the ethics committee of Okayama University.

### Flow cytometry analysis

Peripheral blood was obtained from retroorbital venous plexus and harvested into tubes containing EDTA. Samples were stained with FITC-CD11b and PE-Gr-1 antibodies at 4°C for 1 h. Next, stained samples were washed with PBS, resuspended in 250 μL PBS and analyzed by the MACSQuant Analyzer 10 (Miltenyi Biotec, Cologne, Germany).

### Immunohistochemistry staining

Tumor tissue section was fixed in formalin solution and embedded in paraffin. Then the sections were boiled in 10-mM sodium citrate buffer (pH 6.0) for 20 min at 120°C in a microwave oven for antigen retrieval. Hydrogen peroxide (3%) in methanol was used to inactivate endogenous peroxidase activity for 10 min. Tissue sections were incubated with the primary antibody in a humidified chamber overnight at 4°C. The sections were washed three times with PBS and incubated with Simple StainTM Mouse MAX-PO(R) (414341F, Nichirei Bioscience, Inc., Tsukiji, Tokyo, Japan). DAB reaction was performed using the DAB substrate kits (425312F, Nichirei Bioscience, Inc.).

### Statistical analysis

The analysis was performed using the GraphPad Prism software (GraphPad Prism 8). Data were presented as mean ± standard deviation (S.D.). Statistical differences were analyzed by one-way or two-way analysis of variance. *P*-Values < 0.05 were considered statistically significant.

## Results

### Nitroxoline suppresses cell viability and induces apoptosis in bladder cancer cells

**Figure 1:**
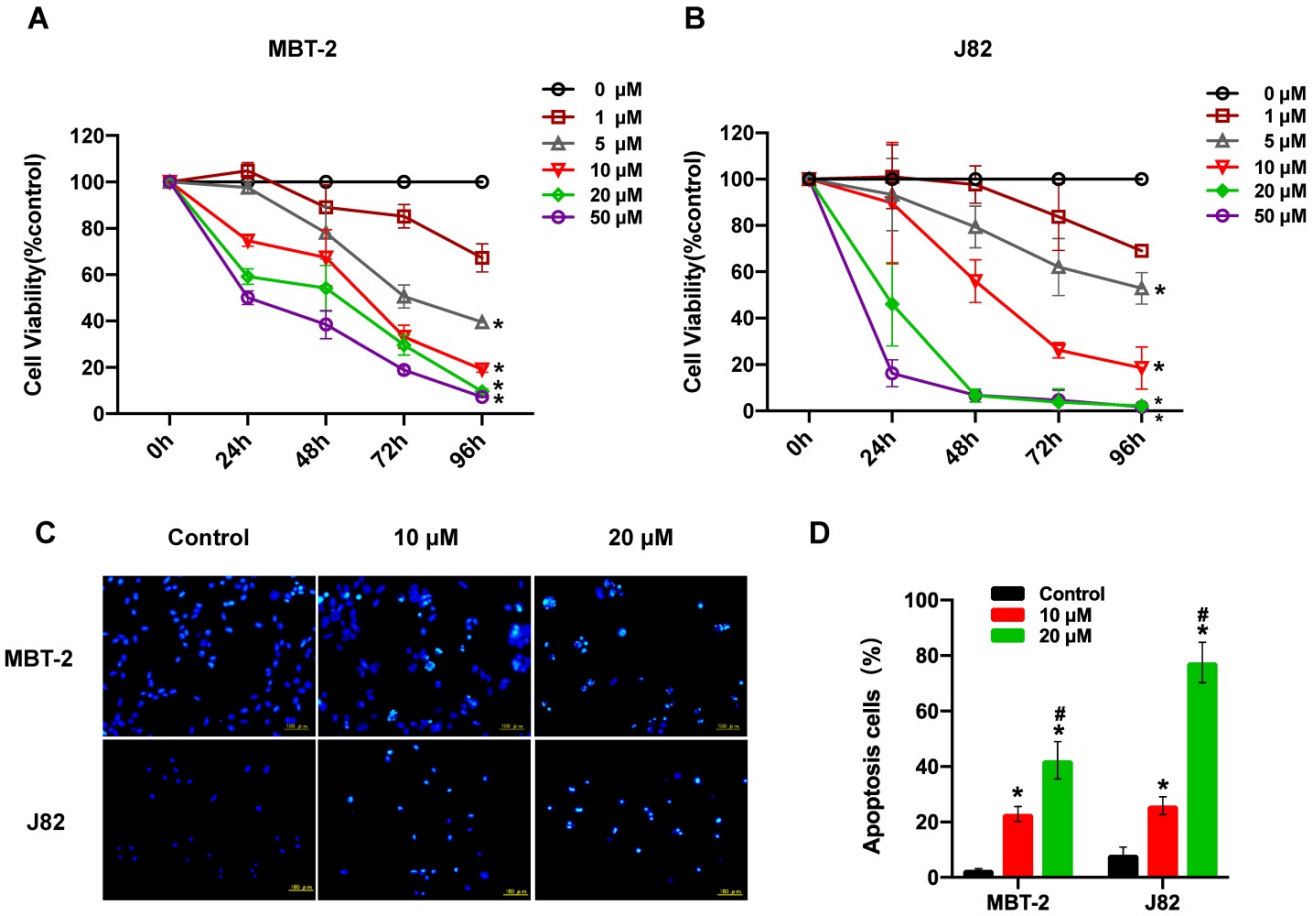
** Nitroxoline suppresses cell viability and induces apoptosis in bladder cancer cells (A and B).** Nitroxoline inhibited bladder cancer cell lines MBT-2, and J82 cell viability in a concentration and time-dependent manner. Data are presented as mean ± S.D. (N = 5, *P < 0.05 vs. 1 μM). (**C**). Hoechst 33324 staining. Bladder cancer cells were treated with nitroxoline for 48h. The obvious apoptotic morphological changes were observed in the nitroxoline-treated group. (**D**). The number of apoptosis cells was quantified by fluorescence microscope. The differences between the nitroxoline treatment group and control group were significant. Data are presented as mean ± S.D. (N = 3, *P < 0.05 vs. control group, **^#^***P* < 0.05 vs. 10μM group).

XTT assay was performed to determine the effect of nitroxoline treatment on the viability of the two bladder cancer cell lines (MBT-2 and J82). Nitroxoline caused the inhibition of bladder cancer cell viability in a concentration (ranging from 1 to 50 µM) and time dependent manner (Fig. 1A and B). Moreover, J82 and MBT-2 exhibited an IC50 of 9.93 μM, 26.24 μM respectively after 48 hours of nitroxoline treatment. J82 cells were more sensitive than MBT-2 to nitroxoline. Hoechst 33324 staining demonstrated that bladder cancer cells treated with 10 or 20 µM nitroxoline for 48h presented obvious apoptotic morphological changes, such as chromatin condensation, nuclear fragmentation and apoptotic bodies (Fig. 1C). In MBT-2 cell, the percentage of apoptotic cells in nitroxoline-treated groups were 22.93±2.68% [10 µM] and 42.30±6.77% [20 µM], compared with 2.77± 0.41% in control group. In J82 cell, the percentage of apoptotic cells in the control group was only 8.18 ± 2.76%, but the percentage increased to 25.92± 3.18% [10 µM] and 77.53± 7.25% [20 µM] in the nitroxoline-treated groups (Fig. 1D).

### Nitroxoline inhibits the migration of bladder cancer cells

**Figure 2:**
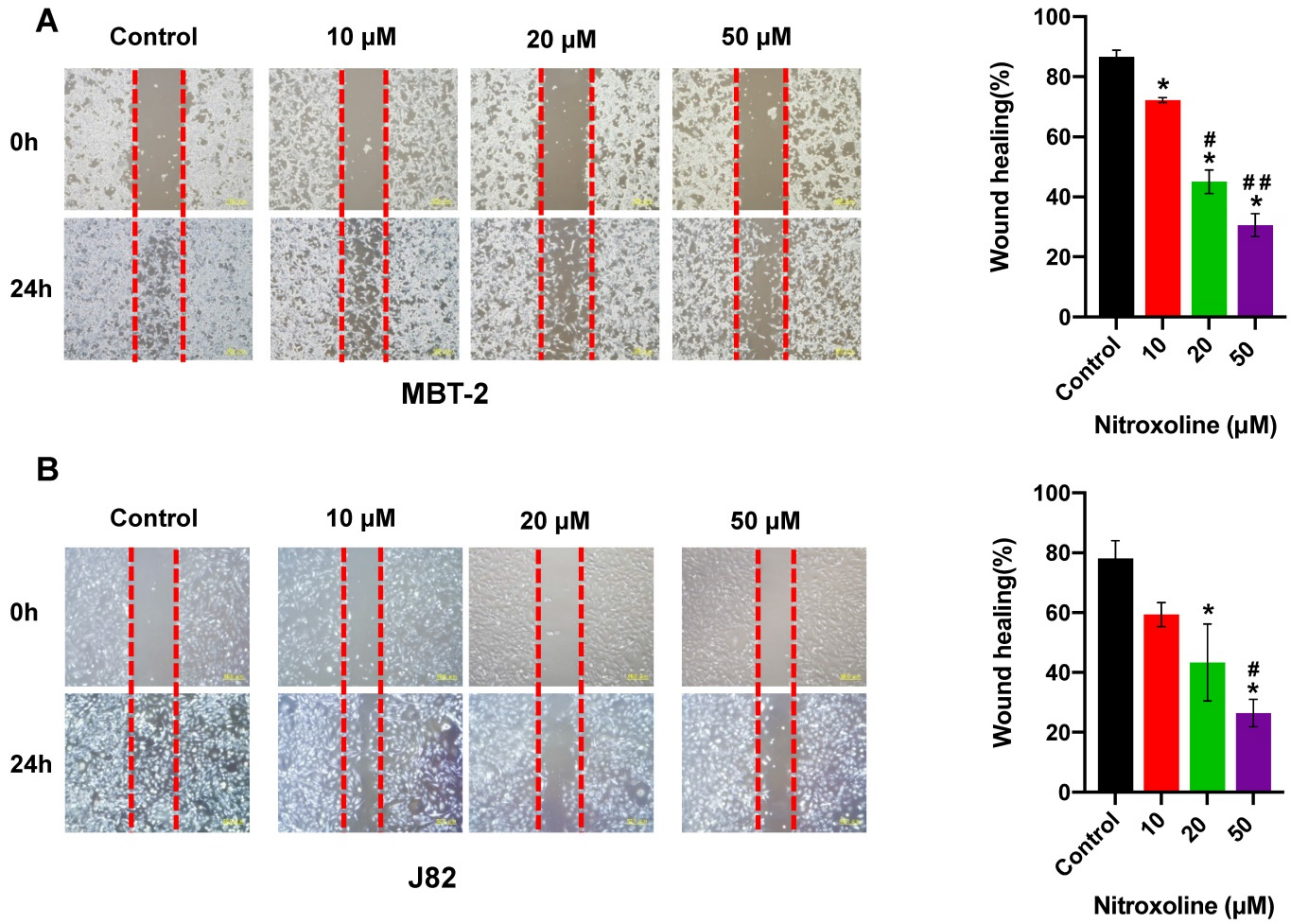

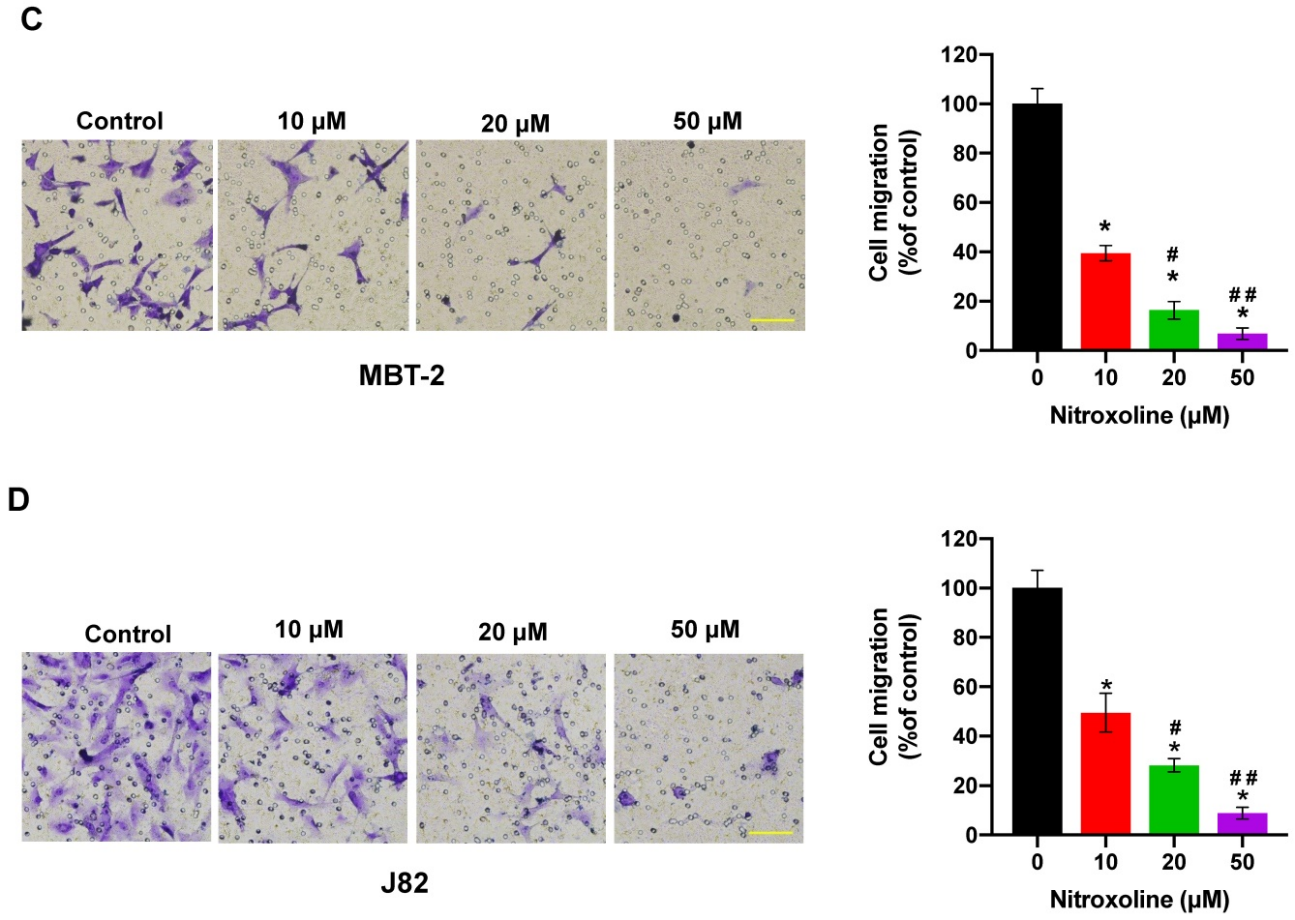
** Nitroxoline inhibits the migration of bladder cancer cells (A and B).** Wound healing assay demonstrated that wound closure was inhibited by nitroxoline in MBT-2 and J82 cells in a concentration-dependent manner. Cell migration was quantified by measuring wound closure areas. Data are presented as the mean ± S.D. (N = 3, *P < 0.05 vs. control group, **^#^***P* < 0.05 vs. 10μM group,**^ ##^***P* < 0.05 vs. 20μM group). **(C and D).** Representative images of transwell migration of nitroxoline-treated bladder cancer cells. Nitroxoline inhibited the migration of MBT-2 and J82 cells. Data are presented as mean ± S.D. Scale bar, 100 μm (N = 3, *P < 0.05 vs. control group, **^#^***P* < 0.05 vs. 10μM group,**^ ##^***P* < 0.05 vs. 20μM group).

To quantify the effect of nitroxoline on cell motility, wound-healing assay was performed using the MBT-2 and J82 cells, and photographed at 0h and 24 h. As shown in Fig. 2A and 2B, bladder cancer cells treated with nitroxoline (ranging from 10 to 50 µM) filled the wound area (area between the two dotted lines) more slowly than those untreated cells, indicating that nitroxoline inhibited cancer cell migration compared to the control group. Similar results were also obtained in transwell assay. The numbers of migrating cells in nitroxoline-treated group were significantly decreased compared with those in the control group (Fig. 2C and D).

### Nitroxoline reverses bladder cancer cells EMT and regulates the expression of apoptosis-related proteins

**Figure 3:**
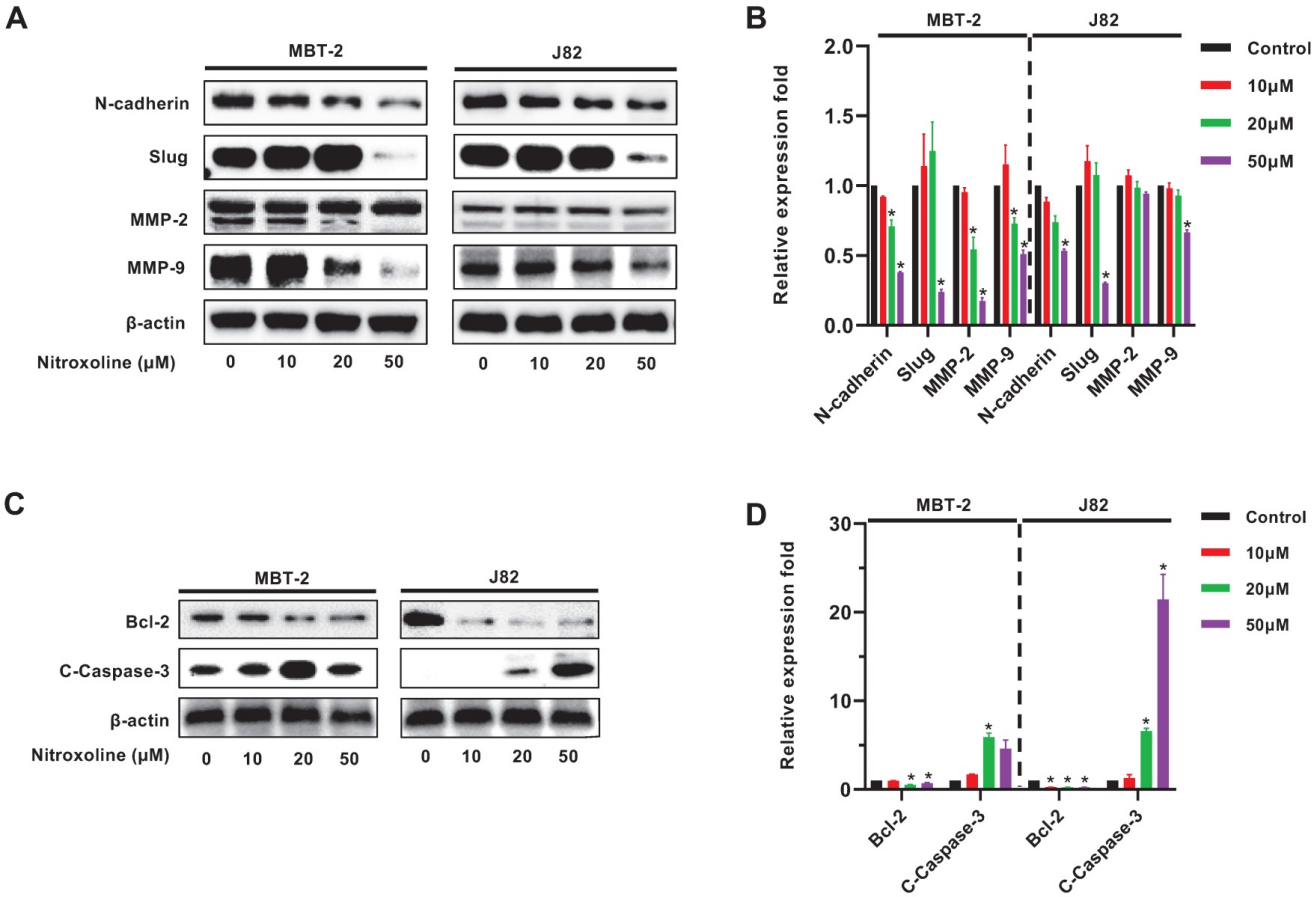
** Western blot analysis of EMT biomarkers and apoptosis related proteins (A).** Bladder cancer cells treated with nitroxoline for 48h expressed reduced levels of N-cadherin, Slug, MMP-2 and MMP-9. (**B**). The expression level of proteins shown in (A) was quantified by ImageJ software, normalized to β-actin, and graphed. (**C**). Nitroxoline decreased the expression of anti-apoptotic protein Bcl-2 and increased the expression of pro-apoptotic protein cleaved caspase-3. (**D**). The expression level of proteins shown in (C) was quantified by ImageJ software, normalized to β-actin, and graphed. β-actin expression was used as an internal control.

Western blot analysis was used to examine expression of EMT-related proteins N-cadherin, slug, MMP-2 and MMP-9 in MBT-2 and J82 cells. The results demonstrated the reduced expression of N-cadherin, slug and MMP-9 in both cell lines, but the MMP-2 expression was decreased only in MBT-2 cells (Fig. 3A and B). In addition, nitroxoline also reduced the expression of the anti-apoptotic protein Bcl-2 as well as elevated the expression of the pro-apoptotic protein cleaved Caspase-3 (C-Caspase-3) (Fig. 3C and D).

### Therapeutic efficacy of nitroxoline against MBT-2 Xenografts

**Figure 4:**
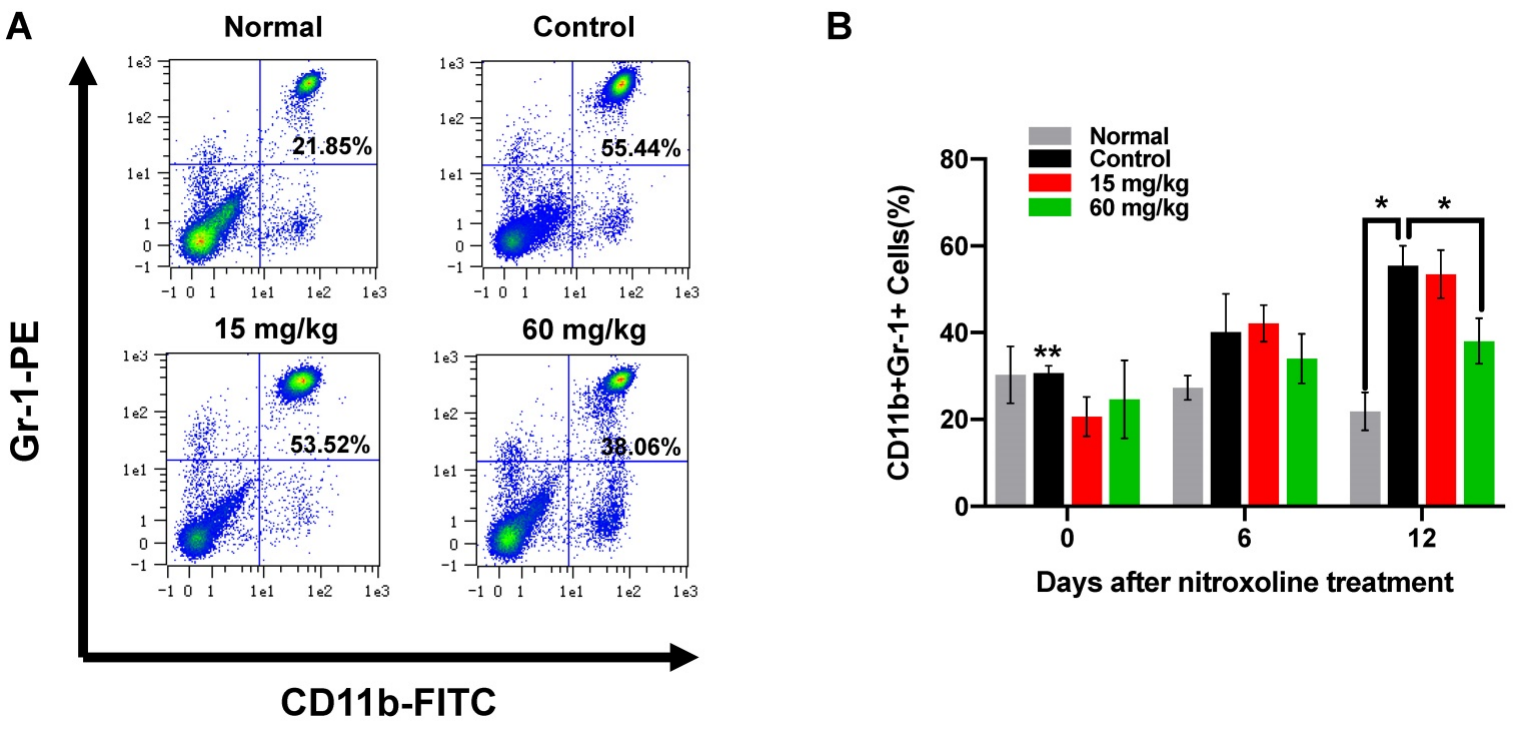
** Nitroxoline inhibits tumor growth in the MBT-2 Xenografts (A).** Tumor growth curves of MBT-2 tumor bearing mice at the indicated days. Data are presented as the mean ± S.D. (N = 7, *P < 0.05). (**B**). Tumors were excised and photographed after 12 days treatment. (**C**). The weight of tumors was significantly decreased in 60mg/kg treatment group, compared with that in control treatment. Data are expressed as the mean ± S.D. (N = 5, *P < 0.05). (**D**). Representative images of immunohistochemical staining for Ki-67, CD31 and MMP-9 in tumor sections from xenografts. All pictures are at the same magnification (200×).

Mice bearing subcutaneous tumors were treated with vehicle or 15mg/kg, 60mg/kg nitroxoline by intragastrically administration for 12 days. As shown in Fig. 4A, high dosage of nitroxoline exhibited suppression of the tumor volume. The tumors weight from 60mg/kg nitroxoline-treated group was statistically significantly lower than that in the vehicle group (0.43±0.20 g vs 1.40±0.53 g; *P* < 0.05) (Fig. 4B and C).

To detect the expressions of Ki-67 and CD31, we performed the immunohistochemistry staining on tissue sections of the resected subcutaneous tumor. 60mg/kg nitroxoline group did exhibit much more serious necrotic regions and less cell proliferation inside their tumor tissues than vehicle group. The expressions of MMP-9 in high dosage group were significantly decreased, compared with vehicle group (Fig. 4D).

### Nitroxoline significantly reduces the percentage of MDSCs in peripheral blood

**Figure 5:**
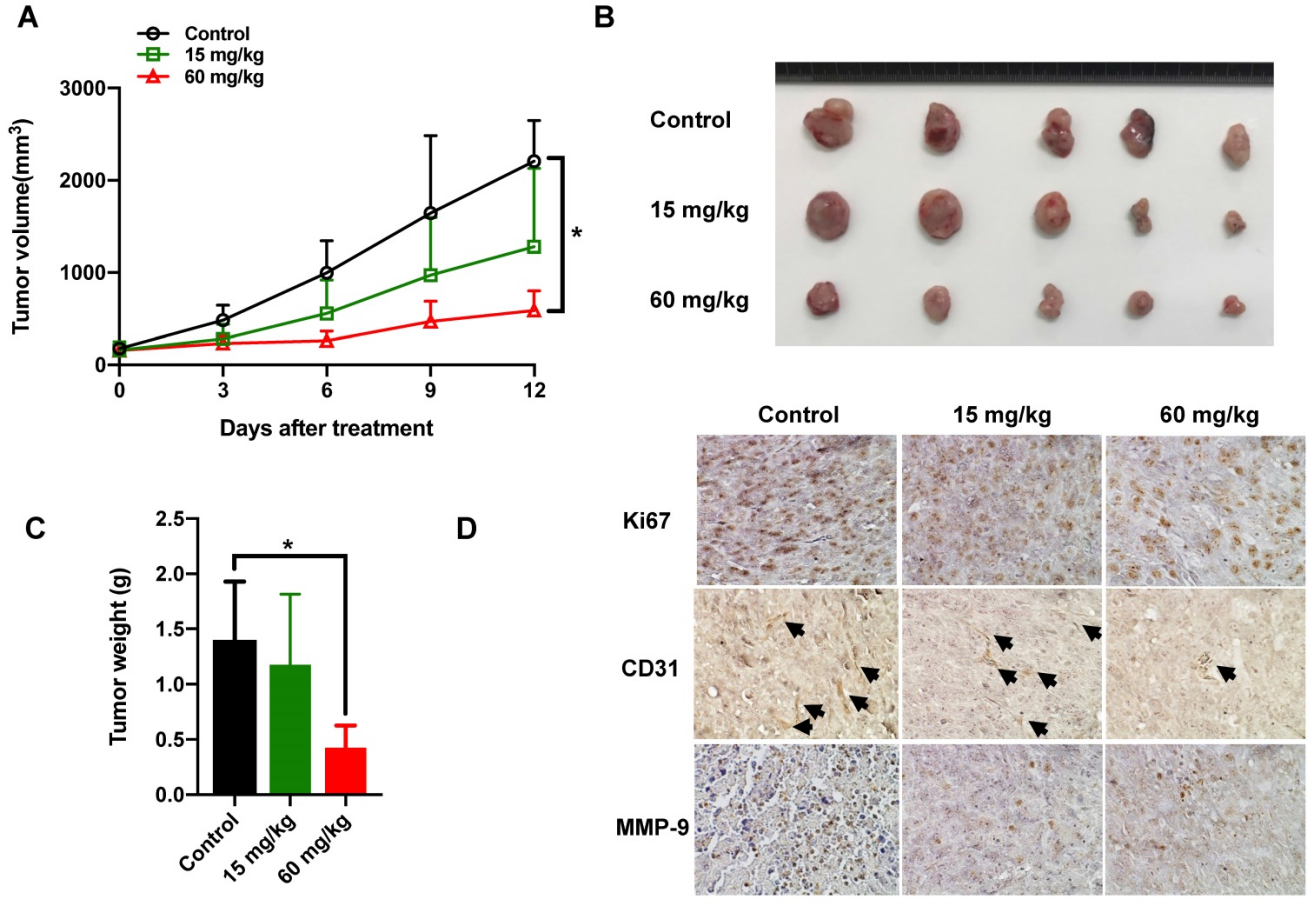
** Nitroxoline significantly reduces the percentage of MDSCs in peripheral blood (A).** Flow cytometric analysis of peripheral blood from tumor-bearing mice as well as normal mice at the experiment endpoint. (**B**). The percentage of CD11b^+^ Gr-1^+^ MDSCs in peripheral blood was quantified by FACS analysis. The differences between 60mg/kg nitroxoline group and control group were significant. Data are presented as mean ± S.D. (N = 3, *P < 0.05, **P < 0.05 vs. control group on day 12).

To determine if nitroxoline treatment inhibits CD11b^+^Gr-1^+^MDSC in C3H/He tumor-bearing mice, mice were treated by gavage with nitroxoline or PBS as a control five times a week on days 1, 2, 4, 5, 7, 8, 10 and 11. In mice, MDSCs are defined as cells co-expressing of the myeloid cell lineage differentiation antigen CD11b and Gr-1. In this study, MDSCs in peripheral blood cells were labelled with FITC-conjugated CD11b and PE-conjugated Gr-1 antibody. MDSCs are a heterogeneous population of myeloid cells that accumulates in many tumor models, and can be divided into two subgroups: granulocytic and monocytic MDSC. The proportion of peripheral blood CD11b^+^Gr-1^+^MDSC was assessed by flow cytometry. During tumor progression, MDSCs were expanded and accumulated in peripheral blood derived from bladder cancer mice model in a time-dependent manner. After the treatment of nitroxoline for 12 days, the percentage of MDSCs were significantly decreased in high dosage of nitroxoline group compared to PBS-treated C3H/He mice (38.06± 5.24% vs. 55.44± 4.58%) (Fig. 5A and B).

## Discussion

In this study, we aimed to reveal the possible mechanisms how nitroxoline inhibits bladder cancer progression. We observed that nitroxoline suppressed cell viability and induced apoptosis in bladder cancer cells. Nitroxoline inhibited the migration of bladder cancer cells. The expressions of epithelial mesenchymal transition (EMT)-related proteins were detected by western blotting. Nitroxoline reversed bladder cancer cells EMT and regulated the expression of apoptosis-related proteins. Using the C3H/He mice of subcutaneous murine bladder cancer (MBT-2) models, we further confirmed that nitroxoline was effective in inhibiting tumor growth. Importantly, nitroxoline significantly reduced the percentage of MDSCs in peripheral blood. Because nitroxoline has been used in the treatment of urinary tract infection in humans, our finding has the potential clinical implication of repurposing it to antitumor application.

It has been reported that nitroxoline results in the suppression of Na/K-ATPase pump and β-catenin, which is closely related with cell growth, migration, invasion, Reactive Oxygen Species (ROS) generation and DNA damage response 5. Our study demonstrated that nitroxoline led to significant inhibition of cell viability and induced apoptosis of bladder cancer cells *in vitro*. In addition, we found a significant downregulation of apoptosis-related protein Bcl-2. Both the Bcl-2 family and caspase family have been identified as critical factors associated with the apoptosis 18. Caspase-3 was examined by western blot analysis and the results showed that cleaved caspase-3 was present, suggesting that caspase-3 was significantly activated after nitroxoline treatment. Some similar studies have shown that nitroxoline can exert an antitumor effect by regulating the expression of caspase-3, PARP, P53, Bcl-xL, and Mcl-1 7, 11, 19. These studies suggest that nitroxoline induced the growth inhibition of bladder cancer cells by the activation of caspase-3, and regulation of Bcl-2 family proteins.

Epithelial-mesenchymal transition (EMT), a process by which tumor epithelial cells acquire the capability to migrate, participates in cell invasion and metastasis 20. It has been known that matrix metalloproteinase-2 (MMP-2) and matrix metalloproteinase-9 (MMP-9) can promote the migration of cancer cells and facilitate extracellular matrix degradation. Cathepsin B is capable of triggering the degradation of tumor stroma and leads to the activation of matrix metalloproteinases zymogens 21. Nitroxoline shows inhibitory activity against cathepsin B and is involved in degradation of extra-cellular membrane proteins 13, 14. Chan-on (2015) reported that nitroxoline significantly suppresses the cell migration and decreases the expression of MMP-2 and MMP-9 in cholangiocarcinoma cells 9. In this study, our results showed that nitroxoline inhibited migration, as determined by wound healing and transwell assay. We further observed the downregulation of EMT markers, namely N-cadherin, Slug, MMP-2 and MMP-9. These findings suggest that nitroxoline caused inhibition of migration of bladder cancer cells via the downregulation of EMT markers, namely N-cadherin, Slug, MMP-2 and MMP-9.

In the model of bladder cancer *in vivo*, we found that nitroxoline treatment (60 mg/kg) exhibited a significant anti-tumor effect. Ki-67 expression is a well-known marker of active proliferation and poor prognosis in bladder cancer. Nitroxoline and its analogues have been proved to have antiangiogenic potential by inhibiting the enzymatic activity of MetAP2 12. CD31 is generally used for the evaluation of angiogenesis and the high expression level implies the activation and proliferation of endothelial cells. In this study, we detected the decreased expressions of Ki-67 and CD31 in tumor tissue. In addition, the altered MMP-9 expressions in local tissues were consistent with the protein level of MMP-9, suggesting that nitroxoline mediated the cell migration through the downregulation of MMP-9. Myeloid‐derived suppressor cells (MDSC) are myeloid cells, negatively affect other immune cells, and particularly result in T-cell exhaustion, leading to immune suppression 22, 23. We demonstrated the decreased levels of MDSC in peripheral blood of tumor-bearing mice after nitroxoline treatment. This indicates that nitroxoline may enhance anti-tumor immunity by decreasing the proportion of MDSC. Further studies are necessary to identify which type of MDSC cell subsets including monocytic MDSC (M-MDSC) and granulocytic MDSC (G-MDSC) responds to nitroxoline and investigate the changes of MDSC in spleen and tumor microenvironment during tumor progression.

In conclusion, we show that nitroxoline suppresses cell viability, migration and induces apoptosis in bladder cancer cells. Moreover, nitroxoline significantly inhibits tumor growth and decreases levels of MDSC *in vivo* tumor mouse models. Considering that nitroxoline are approved by the FDA, our findings suggest that nitroxoline may be a potential candidate for bladder cancer.

## Abbreviations

MDSCs: myeloid- derived suppressor cells
EMT: epithelial mesenchymal transition
FDA: Food and Drug Administration
